# Effect of Cardiovascular Risk Factors on 30-Day All-Cause Mortality in Cardiogenic Shock

**DOI:** 10.3390/jcm12144870

**Published:** 2023-07-24

**Authors:** Jan Forner, Tobias Schupp, Kathrin Weidner, Marinela Ruka, Sascha Egner-Walter, Michael Behnes, Muharrem Akin, Mohamed Ayoub, Kambis Mashayekhi, Ibrahim Akin, Jonas Rusnak

**Affiliations:** 1 Department of Cardiology, Angiology, Haemostaseology and Medical Intensive Care, University Medical Centre Mannheim, Medical Faculty Mannheim, Heidelberg University, 68167 Mannheim, Germany; se183@uni-heidelberg.de (J.F.);; 2 European Center for AngioScience (ECAS), German Center for Cardiovascular Research (DZHK) Partner Site Heidelberg/Mannheim, 68167 Mannheim, Germany; 3 Department of Cardiology and Angiology, Hannover Medical School, 30625 Hannover, Germany; 4 Division of Cardiology and Angiology, Heart Center, University of Bochum, 32545 Bad Oeynhausen, Germany; ayoub@hotmail.de; 5 Department of Internal Medicine and Cardiology, Mediclin Heart Centre Lahr, 77933 Lahr, Germany

**Keywords:** cardiogenic shock, cardiovascular risk factors, prognosis, mortality

## Abstract

Although previous studies investigated the influence of cardiovascular risk (CVR) factors in patients with acute coronary syndrome, data concerning the effect of CVR factors on the prognosis of patients with cardiogenic shock (CS) is scarce. Consecutive patients with CS were prospectively included from 2019 to 2021. The prognosis of patients with “low CVR” (i.e., 0–1 CVR factors) was compared to patients with “high CVR” (i.e., 2–4 CVR factors) according to presence or absence of arterial hypertension, diabetes mellitus, hyperlipidaemia or smoking. The primary endpoint was 30-day all-cause mortality. Statistical analyses included Kaplan-Meier and Cox proportional regression analyses. 273 consecutive patients with CS were included. 28% presented with low CVR and 72% with high CVR. Within the entire study cohort, the risk of 30-day all-cause mortality did not differ between patients with high and low CVR (55% vs. 57%; log rank *p* = 0.727; HR = 0.942; 95% CI 0.663–1.338; *p* = 0.738). Even after multivariable adjustment, high CVR was not associated with an elevated risk of 30-day all-cause mortality (HR = 1.039; 95% CI 0.648–1.667; *p* = 0.873). The presence of arterial hypertension (55% vs. 58%; log rank *p* = 0.564; HR = 0.906; 95% CI 0.638–1.287; *p* = 0.582), diabetes mellitus (60% vs. 52%; log rank *p* = 0.215; HR = 1.213; 95% CI 0.881–1.671; *p* = 0.237) and a history of smoking (56% vs. 56%; log rank *p* = 0.725; HR = 0.945; 95% CI 0.679–1.315; *p* = 0.737) did not significantly influence short-term prognosis.. Only the absence of hyperlipidaemia significantly decreased the risk of all-cause mortality (65% vs. 51%; log rank *p* = 0.038; HR = 0.718; 95% CI 0.516–0.998; *p* = 0.049), which was no longer observed after multivariable adjustment (HR = 0.801; 95% CI 0.536–1.195; *p* = 0.277). In conclusion, neither the overall CVR nor individual CVR factors were associated with the risk of 30-day all-cause mortality in patients with CS.

## 1. Introduction

Cardiogenic shock (CS) is a subtype of shock in which a primarily cardiac pathology causes a mismatch between blood supply of the heart and demanded tissue perfusion. In the majority of cases, this inability to provide sufficient circulation originates in substantial myocardial ischaemia [1]. Accordingly, CS is a meaningful complication in patients with acute myocardial infarction (AMI), occurring in up to 13% of the patients with ST-elevation myocardial infarction (STEMI) [2,3]. Despite early revascularization strategies and the use of mechanical circulatory support devices, CS-related mortality remains dissatisfying with some authors even indicating a rise of in-hospital mortality rates in patients with myocardial infarction complicated by CS undergoing percutaneous coronary intervention (PCI) [4,5]. Therefore, comprehending which biomarker and risk factor may or may not disclose CS patients with greater risk of dying is of utmost importance.

Cardiovascular risk (CVR) factors, particularly arterial hypertension, diabetes mellitus, hyperlipidaemia, as well as smoking, are the main drivers of atherosclerosis, and thus, cardiovascular illnesses like coronary artery disease (CAD) [6]. As such, those so-called standard modifiable risk factors (SMuRFs) are accountable for the majority of global deaths, according to the “Global Burden of Disease Study 2017” [7,8]. Consequently, reducing SMuRFs is a pivotal role in current cardiologic guidelines [6].

While a few studies focusing on the prognostic outcome of SMuRFs in patients with acute coronary syndrome (ACS) are available, to the best of the authors knowledge, no studies investigating the influence of SMuRFs in patients with CS were published yet [9,10,11,12,13,14]. Therefore, this study aims to analyse the prognostic value of SMuRFs by comparing CS patients with high (i.e., 2–4 CVR factors) vs. low CVR (i.e., 0–1 CVR factors) with regard to 30-day all-cause mortality. Thereafter, the prognostic impact of the individual SMuRFs on short-term mortality was tested.

## 2. Materials and Methods

### 2.1. Study Patients, Design and Data Collection

This study prospectively included all consecutive CS patients admitted to the internistic intensive care unit (ICU) at the University Medical Center Mannheim, Germany, from June 2019 to May 2021. Clinically relevant data related to the index event was documented using the electronic hospital information system as well as the IntelliSpace Critical Care and anaesthesia information system (ICCA, Philips, Philips GmbH Market DACH, Hamburg, Germany), which are implemented at the ICU to organize patient data (i.e., admission documents, vital signs, laboratory values, treatment data and consult notes).

Laboratory parameters, ICU-related scores, hemodynamic measurements and ventilation parameters of the day of admission (i.e., day 1), as well as of days 2, 3, 4 and 8 were collected. Moreover, data concerning prior medical history, pharmacological therapy and medical imaging was documented as well as the duration of index hospital stay. The source data was recorded by intensivists and ICU nurses during routine clinical care.

This study derives from an analysis of the “Cardiogenic Shock Registry Mannheim” (CARESMA-registry). The CARESMA-registry represents a prospective single-center registry including consecutive CS patients admitted to the ICU for internal medicine of the University Medical Center Mannheim (UMM), Germany (clinicaltrials.gov identifier: NCT05575856), as recently published [15]. The registry was established according to the principles of the declaration of Helsinki and was approved by the medical ethics committee II of the Medical Faculty Mannheim, University of Heidelberg, Germany.

### 2.2. Inclusion and Exclusion Criteria, Study Endpoints

For this study, all consecutive patients with CS were included. The diagnosis of CS was made in accordance with current recommendations of the Acute Cardiovascular Care Association of the European Society of Cardiology [1]. Correspondingly, cardiogenic shock was defined as hypotension (SBP < 90 mmHg) for more than 30 min despite adequate filling status or need for vasopressor or inotropic therapy to achieve SBP > 90 mmHg. Additionally, signs for end-organ hypoperfusion, such as oliguria with urine output < 30 mL/hour, altered mental status, cold clammy skin, or increased serum lactate > 2 mmol/L had to be present. Patients with “low CVR” (i.e., 0–1 CVR factors) were compared to patients with “high CVR” (i.e., 2–4 CVR factors). CVR factors included the presence of arterial hypertension, diabetes mellitus, hyperlipidaemia or smoking. The presence or absence of CVRs was assessed according to first diagnosis during index hospitalization or prior medical history. Arterial hypertension was defined by systolic blood pressure levels ≥ 140 mmHg and/or diastolic blood pressure levels ≥ 90 mmHg, causing the benefits of treatment (i.e., lifestyle interventions or medication) to be superior to the risks [16]. Diabetes mellitus was defined in the presence of glycated haemoglobin A_1c_ (HbA_1c_) ≥ 6.5%, fasting plasma glucose levels ≥ 126 mg/dL or 2-h post-load plasma glucose levels ≥ 200 mg/dL in accordance with established guidelines [17]. Hyperlipidaemia was determined as previously diagnosed hyperlipidaemia, prescribed lipid-lowering therapy or serum low-density lipoprotein (LDL) levels > 115 mg/dL during index hospitalisation [18]. Patients were characterised as smokers if they were ex- or current smokers correspondent to prior or latest anamnesis. No exclusion criteria were applied for the present study.

The primary endpoint was all-cause mortality at 30-days. All-cause mortality was recorded using the electronic hospital information system and by directly contacting state resident registration offices (‘bureau of mortality statistics’). The patients’ identity was verified by place of name, surname, day of birth, as well as registered living address. No patient was lost to follow-up with respect to all-cause mortality at 30 days.

### 2.3. Statistical Methods

Quantitative- data is presented as median and interquartile range (IQR). Student’s t test and the Mann-Whitney U test were used to compare normally distributed and nonparametric data, respectively. The Kolmogorov-Smirnov test was applied to test for deviations from a Gaussian distribution. Qualitative data is presented in form of absolute and relative frequencies and was compared using the Chi-square test or Fisher’s exact test, as appropriate. Kaplan-Meier analyses were performed to compare the prognostic outcome of high CVR vs. low CVR patients with regard to the primary endpoint (i.e., all-cause mortality at 30-days). Thereafter, Kaplan-Meier analyses were used to analyse the prognostic impact of the individual CVR factors on the risk of 30-day all-cause mortality. Univariable hazard ratios (HR) were given together with 95% confidence intervals. Finally, multivariable Cox regression models were developed using the “forward selection” option.

Results of all statistical tests were considered significant for *p* ≤ 0.05. SPSS (Version 28, IBM, Armonk, New York, NY, USA) was used for statistics.

## 3. Results

### 3.1. Study Population

273 patients with CS were admitted to our institution from 2019 to 2021. Within the entire study cohort, 197 (72%) patients had concomitant arterial hypertension, 110 (40%) patients had diabetes mellitus, 187 (69%) patients had hyperlipidaemia and 99 (36%) patients were smokers. The median age was 73 years and most of the patients were males (60%) (Table 1; left panel). Overall, 56% (*n* = 152) of the patients reached the primary endpoint of 30-day all-cause mortality (Table 2; left panel). As seen in Table 1, non-survivors presented with lower body temperatures (median 35.8 vs. 36.1 °C; *p* = 0.033) and higher heart rates (median 94 vs. 85 bpm; *p* = 0.049) than survivors. Except for hyperlipidaemia, which was more frequent in survivors (75% vs. 63%; *p* = 0.033), cardiovascular risk factors were evenly distributed between survivors and non-survivors. Moreover, the prior medical history and medication on admission differed not significantly between both groups. As displayed in Table 2, AMI, as a cause of CS, was significantly more common in non-survivors compared to survivors (57% vs. 40%; *p* = 0.005), whereas an arrhythmic cause was more frequent in survivors than in non-survivors (21% vs. 5%; *p* = 0.001). Both out-of-hospital cardiac arrest (44% vs. 31%; *p* = 0.022) and in-hospital cardiac arrest (22% vs. 11%; *p* = 0.011) were observed more often in non-survivors than in survivors. Furthermore, non-survivors required mechanical ventilation (63% vs. 50%; *p* = 0.024) and mechanical circulatory assist devices (13% vs. 4%; *p* = 0.010) more frequently compared to survivors. Regarding baselines laboratory values, non-survivors especially showed higher lactate levels (4.7 vs. 2.6 mmol/L; *p* = 0.001), higher creatinine levels (1.59 vs. 1.31 mg/dL; *p* = 0.006), higher triglyceride levels (108 vs. 94 mg/dL; *p* = 0.026) and higher troponin I levels (1.850 vs. 0.332 µg/L; *p* = 0.001) than survivors. In contrast, survivors had significantly higher albumin levels compared to non-survivors (30.7 vs. 28.7 g/L; *p* = 0.005). Baselines characteristics and shock-related data comparing patients with low CVR to patients with high CVR are outlined in Supplemental Tables S1 and S2. Of note, patients with low CVR were more likely to have out-of-hospital cardiac arrest compared to high CVR patients (56% vs. 31%; *p* = 0.001).

### 3.2. Prognostic Impact of the CVR

**Figure 1:**
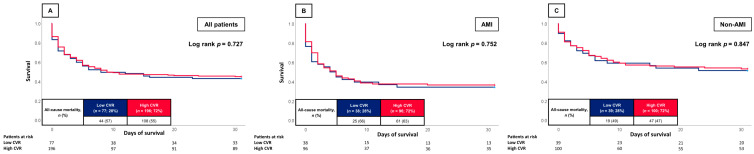
Kaplan-Meier curves for low and high CVR within the entire study cohort (**A**), patients with AMI (**B**) and non-AMI patients (**C**).

At 30 days, the primary endpoint of all-cause mortality occurred in 55% of the patients with high CVR and 57% of the patients with low CVR. Accordingly, the risk of all-cause mortality was not affected by the CVR (log rank *p* = 0.727; HR = 0.942; 95% CI 0.663–1.338; *p* = 0.738) (Figure 1A). Furthermore, no association between the CVR and short-term all-cause mortality was found when stratified for AMI- (63% vs. 66%; log rank *p* = 0.752; HR = 0.932; 95% CI 0.585–1.485; *p* = 0.767) and non-AMI-related CS (47% vs. 49%; log rank *p* = 0.847; HR = 0.950; 95% CI 0.558–1.619; *p* = 0.851) (Figure 1B,C). Even after multivariable adjustment, high CVR was not associated with an increased risk of 30-day all-cause mortality (HR = 1.039; 95% CI 0.648–1.667; *p* = 0.873). Instead, high lactate levels (HR = 1.134; 95% CI 1.087–1.183; *p* = 0.001) and the presence of AMI (HR = 2.089; 95% CI 1.205–3.620; *p* = 0.009) were independently associated with an elevated risk of 30-day all-cause mortality (Table 3). Comparing patients with 0 vs. 1–4 (61% vs. 55%; log rank *p* = 0.407; HR = 0.811; 95% CI 0.483–1.362; *p* = 0.429) or 0–2 vs. 3–4 (58% vs. 53%; log rank *p* = 0.310; HR = 0.852; 95% CI 0.616–1.178; *p* = 0.333) CVR factors resulted in comparable outcomes with no statistically significant association between, 30-day all-cause mortality and different CVR.

**Figure 2:**
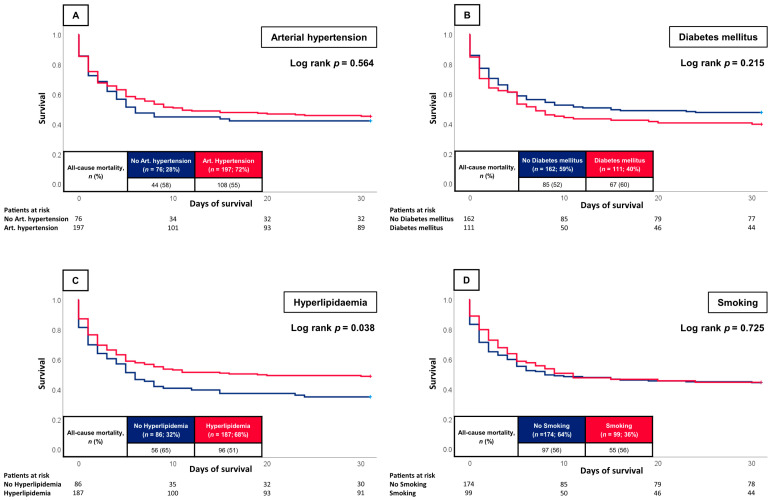
Kaplan-Meier curves for arterial hypertension (**A**) diabetes mellitus (**B**), hyperlipidaemia (**C**) and smoking (**D**).

**Figure 3:**
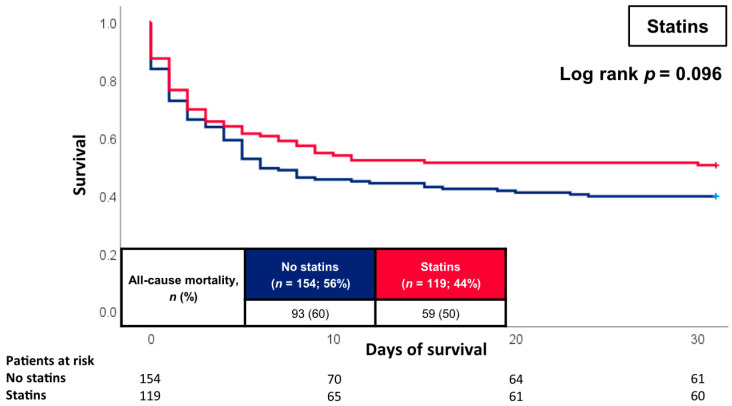
Kaplan-Meier curves for the prescription of statins.

Figure 2 illustrates the prognostic impact of individual CVR factors on 30-day all-cause mortality. The primary endpoint of 30-day all-cause mortality was reached by 55% of the patients with and 58% of the patients without arterial hypertension. Hence, the presence of arterial hypertension did not significantly heighten the risk of short-term mortality (log rank *p* = 0.564; HR = 0.906; 95% CI 0.638–1.287; *p* = 0.582). Likewise, neither the presence of diabetes mellitus (60% vs. 52%; log rank *p* = 0.215; HR = 1.213; 95% CI 0.881–1.671; *p* = 0.237) nor a history of smoking (56% vs. 56%; log rank *p* = 0.725; HR = 0.945; 95% CI 0.679–1.315; *p* = 0.737) significantly influenced the risk of 30-day all-cause mortality. In contrast, the absence of hyperlipidaemia displayed a statistically significant association with the primary endpoint compared to patients with hyperlipidaemia (65% vs. 51%; log rank *p* = 0.038; HR = 0.718; 95% CI 0.516–0.998; *p* = 0.049). Patients with statins had comparable risk of 30-day all-cause mortality compared to patients without the prescription of statins (50% vs. 60%; log rank *p* = 0.096; HR = 0.768; 95% CI 0.554–1.064; *p* = 0.113) (Figure 3). After multivariable Cox regression analyses, none of the individual CVR factors, i.e., hyperlipidaemia (HR = 0.801; 95% CI 0.536–1.195; *p* = 0.277), arterial hypertension (HR = 0.679; 95% CI 0.433–1.065; *p* = 0.092), diabetes mellitus (HR = 1.112; 95% CI 0.751–1.646; *p* = 0.596) and smoking (HR = 1.292; 95% CI 0.872–1.913; *p* = 0.202), presented as independent risk factors with regard to 30-day all-cause mortality (Table 4). Yet, related to the model used in Table 3, high lactate levels (HR = 1.119; 95% CI 1.079–1.162; *p* = 0.001), the presence of AMI (HR = 1.863; 95% CI 1.286–2.697; *p* = 0.001) as well as increasing age (HR = 1.016; 95% CI 1.000–1.032; *p* = 0.048) were independently associated with 30-day all-cause mortality.

## 4. Discussion

This study investigates the prognostic impact of SMuRFs on 30-day all-cause mortality in consecutive patients suffering from CS at an internistic ICU. The study suggests, that patients with high CVR have no increased risk of 30-day all-cause mortality compared to patients with low CVR. Moreover, except for hyperlipidaemia, none of the SMuRFs showed a significant effect on short-term mortality. In fact, the presence of hyperlipidaemia decreased the risk of short-term mortality, though this association was no longer observed after multivariate adjustment. These results deliver comprehensive information about the role of SMuRFs within a large study group of CS patients admitted to an internistic ICU.

As aforementioned, recent studies have examined the role of SMuRFs in patients presenting with ACS. Typically, those studies compare patients with at least one of the SMuRFs (i.e., arterial hypertension, hyperlipidaemia, smoking or diabetes mellitus) to patients without SMuRFs (i.e., SMuRF-less patients). Firstly, in 2017, Vernon et al. [19] hypothesized a change of the amount of SMuRF-less STEMI patients. By examining 536 STEMI patients within a single centre retrospective study, they observed an increase of SMuRF-less STEMI patients between 2006 and 2014 from 11% to 27%. However, no difference in age, history or extent of CAD, length of hospital stay, in-hospital mortality or cardiovascular discharge medication regime was found. Later, this study group conducted a retrospective analysis of 62,048 patients with first-presentation STEMI using data of the SWEDEHEART national registry [20]. They described SMuRF-less patients as presenting with lower body mass indices (BMI) and less chronic cardiovascular diseases. Nonetheless, SMuRF-less patients were at higher risk of in-hospital and 30-day all-cause mortality as well as CS. Furthermore, SMuRF-less patients were less likely to receive guideline-recommended medication (i.e., angiotensin converting enzyme inhibitors, angiotensin receptor blockers, β-blockers or statins) at discharge. Interestingly, the excess all-cause mortality was, despite decline after 30 days, still evident after long-term follow-up and was attenuated after adjusting for the use of guideline-oriented medication. These findings are in line with a global meta-analysis of 15 studies including 1,285,722 patients with STEMI or non-ST-elevation acute coronary syndrome (NSTE-ACS) by Kong et al. [11], demonstrating higher risks of all-cause in-hospital mortality, in-hospital cardiac death and CS as well as lower rates of prior heart failure, chronic kidney disease or stroke in SMuRF-less patients. Yet, a prospective cohort study by Moledina et al. [21], including 176,083 patients with non-ST-elevation myocardial infarction (NSTEMI) from the United Kingdom myocardial infarction national audit project (MINAP), found decreased risks of in-hospital all-cause mortality, cardiac mortality and major adverse cardiovascular events (MACE) in patients without SMuRFs compared to patients with SMuRFs after multivariable adjustment. Considering baseline characteristics, the results of the low CVR group of our study are comparable with current literature, in that SMuRF-less patients are less likely to present with a prior history of heart failure and stroke [11,12,13,20]. However, a major difference of our study is, that contrary to literature describing impaired short-term prognosis of patients without SMuRFs [9,10,11,12,13,14,20], 30-day all-cause mortality of patients with low and high CVR was comparable in our study.

There are several possible explanations for these observations. Firstly, unlike our studies, previous studies were not restricted to patients with CS. Thus, patients included in our study were already at a much higher risk of death compared to studies including all patients with AMI. For instance, Figtree et al. [20] reported a 30-day all-cause mortality of 11.3% and 7.9% in SMuRF-less patients compared to patients with one or more SMuRF, respectively. In our study, 30-day all-cause mortality was as high as 57% in low CVR patients and 55% in high CVR patients. Therefore, this study introduces the notion, that, even though CS occurs more frequent in SMuRF-less patients with AMI [9,11,12,13,14,20], in CS itself, CVR does not determine prognosis. Secondly, several authors discovered a deficiency in guideline-oriented medication at discharge in SMuRF-less patients [11,20,22]. Since CS is an illness with severely impaired prognosis, clinicians may be more inclined to prescribe guideline-indicated medication at discharge even to patients with low CVR.

Cardiac arrest is a feared complication of CS. Concurrent with present studies showing higher rates of cardiac arrest in SMuRF-less patients [9,11,12,14,20], in our study, patients with low CVR presented more often with out-of-hospital cardiac arrest (OHCA) than patients with high CVR. According to current knowledge, the principal proportion of sudden cardiac death (SCD) is made up of patients with concealed cardiovascular disease [23]. Thus, the elevated prevalence of OHCA in low CVR patients may be attributed to missed diagnosis of cardiovascular diseases due to the lack of SMuRFs. Furthermore, since ventricular arrhythmias typically precede OHCA [24], our findings support the hypothesis that patients with low CVR are at higher risk for developing arrhythmias as proposed by several authors investigating in the impact of SMuRFs on patients with ACS or AMI [11,13,20]. Overall, this emphasizes the need for novel parameters independent of SMuRFs to identify patients at risk for adverse cardiovascular events [11,12,13,19,20].

In this study, except for hyperlipidaemia, none of the SMuRFs displayed a significant effect on short-term mortality in Kaplan-Meier analysis. These results are in line with a previously published work of our study group, paradoxically revealing no significant impact of arterial hypertension or smoking on long-term survival in patients with ventricular tachyarrhythmias [25].

The decreased risk of short-term all-cause mortality in patients with hyperlipidaemia in our study may be attributed to the prescription of statins in this group, which exhibit several positive effects besides decreasing LDL levels, such as increasing plaque stability, and have been shown to improve survival in varying clinical conditions [26,27]. Albeit, in this study, the risk of 30-day all-cause mortality between patients with and without statins was comparable. These results are in line with previous results of our study group, showing no significant impact of statin therapy on long-term outcomes in patients with electrical storm [28], and may also be related to the already heavily compromised prognosis in both patients with CS and electrical storm.

## 5. Limitations

The present study has several limitations. Despite adjustment for potential confounders using multivariable Cox regression analyses, results may be affected by measured and unmeasured confounding due to the monocentric and observational study design. Furthermore, some selection bias related to difficulties with retrieving data about prior diagnoses in high-risk patients with CS may still be present despite the prospective study design. Finally, associations of CVR factors with long-term outcomes were beyond the scope of this study.

## 6. Conclusions

In conclusion, this study revealed, that neither the overall CVR nor individual CVR factors determine short-term prognosis in patients admitted with CS. Thus, clinicians ought to be cautious even when treating patient with CS and supposedly “low CVR”.

## Figures and Tables

**Table 1:** Baseline characteristics.

	All Patients (*n* = 273)		Survivors (*n* = 121)		Non-Survivors (*n* = 152)		*p* Value
**Age**, median; (IQR)	73	(63–81)	72	(62–79)	74	(64–81)	0.152
**Male sex**, *n* (%)	164	(60.1)	73	(60.3)	91	(59.9)	0.938
**Body mass index**, kg/m^2^ (median, (IQR))	26.3	(24.2–30.0)	26.1	(23.9–28.0)	26.7	(24.5–30.5)	0.111
**Entry criteria**, (median, (IQR))							
Body temperature (°C)	36.0	(35.0–36.6)	36.1	(35.3–36.6)	35.8	(34.7–36.5)	**0.033**
Heart rate (bpm)	88	(71–109)	85	(69–105)	94	(72–111)	**0.049**
Systolic blood pressure (mmHg)	109	(92–130)	110	(94–131)	106	(89–127)	0.228
Respiratory rate (breaths/min)	20	(17–24)	19	(16–22)	20	(18–25)	0.066
**Cardiovascular risk factors**, *n* (%)							
Arterial hypertension	197	(72.2)	89	(73.6)	108	(71.1)	0.647
Diabetes mellitus Type 1	2	(0.7)	0	(0.0)	2	(1.3)	0.205
Diabetes mellitus Type 2	108	(39.6)	44	(36.4)	64	(42.1)	0.335
Hyperlipidaemia	187	(68.5)	91	(75.2)	96	(63.2)	**0.033**
Smoking	99	(36.3)	44	(36.4)	55	(36.2)	0.976
**Prior medical history**, *n* (%)							
Coronary artery disease	101	(37.0)	44	(36.4)	57	(37.5)	0.847
Congestive heart failure	96	(35.2)	43	(35.5)	53	(34.9)	0.908
Atrial fibrillation	87	(31.9)	39	(32.2)	48	(31.6)	0.909
Chronic kidney disease	93	(34.1)	41	(33.9)	52	(34.2)	0.955
Stroke	37	(13.6)	21	(17.4)	16	(10.5)	0.102
COPD	52	(19.0)	19	(15.7)	33	(21.7)	0.209
Liver cirrhosis	9	(3.3)	6	(5.0)	3	(2.0)	0.170
**Medication on admission**, *n* (%)							
ACE-inhibitor	92	(33.7)	43	(35.5)	49	(32.2)	0.567
ARB	48	(17.6)	22	(18.2)	26	(17.1)	0.816
Beta-blocker	135	(49.5)	63	(52.1)	72	(47.4)	0.441
ARNI	8	(2.9)	5	(4.1)	3	(2.0)	0.293
Aldosterone antagonist	41	(15.0)	19	(15.7)	22	(14.5)	0.778
Diuretics	117	(42.9)	48	(39.7)	69	(45.4)	0.342
ASA	78	(28.6)	33	(27.3)	45	(29.6)	0.672
P2Y12-inhibitor	23	(8.4)	10	(8.3)	13	(8.6)	0.932
Statin	119	(43.6)	60	(49.6)	59	(38.8)	0.075
Metformin	31	(11.4)	15	(12.4)	16	(10.5)	0.628
Sulfonylureas	3	(1.1)	3	(2.5)	0	(0.0)	0.051
GLP-1-RA	4	(1.5)	2	(1.7)	2	(1.3)	0.818
DPP-4-inhibitors	36	(13.2)	16	(13.2)	20	(13.2)	0.987
SGLT2-inhibitors	10	(3.7)	4	(3.3)	6	(3.9)	0.779
Insulin	47	(17.2)	20	(16.5)	27	(17.8)	0.788

ACE, angiotensin-converting-enzyme; ARB angiotensin receptor blocker; ARNI, angiotensin receptor neprilysin inhibitor; ASA, acetylsalicylic acid; COPD, chronic obstructive pulmonary disease; CVR, cardiovascular risk; DPP-4-inhibitors, dipeptidyl peptidase-4 inhibitors; GLP-1-RA, glucagon-like peptide-1 receptor agonists; IQR, interquartile range; SGLT2-inhibitors, sodium-glucose cotransporter-2 inhibitors. Level of significance *p* < 0.05.

**Table 2:** Shock-related data, follow-up data and endpoints.

	All Patients (*n* = 273)		Survivors (*n* = 121)		Non-Survivors (*n* = 152)		*p* Value
**Cause of CS**, *n* (%)							
Acute myocardial infarction	134	(49.1)	48	(39.7)	86	(56.6)	**0.005**
Arrhythmic	32	(11.7)	25	(20.7)	7	(4.6)	**0.001**
ADHF	67	(24.5)	27	(22.3)	40	(26.3)	0.445
Pulmonary embolism	15	(5.5)	4	(3.3)	11	(7.2)	0.157
Valvular	12	(4.4)	8	(6.6)	4	(2.6)	0.111
Cardiomyopathy	7	(2.6)	4	(3.3)	3	(2.0)	0.489
Pericardial tamponade	5	(1.8)	5	(4.1)	0	(0.0)	**0.011**
Aortic dissection	1	(0.4)	0	(0.0)	1	(0.7)	0.371
**Classification of CS**, *n* (%)							
Stage A	0	(0.0)	0	(0.0)	0	(0.0)	-
Stage B	6	(2.2)	6	(5.0)	0	(0.0)	**0.006**
Stage C	96	(35.2)	56	(46.3)	40	(26.3)	**0.001**
Stage D	20	(7.3)	9	(7.4)	11	(7.2)	0.949
Stage E	151	(55.3)	50	(41.3)	101	(66.4)	**0.001**
**Transthoracic echocardiography**							
LVEF > 55%, (*n*, %)	28	(11.2)	16	(13.8)	12	(9.0)	0.226
LVEF 54–41%, (*n*, %)	32	(12.8)	21	(18.1)	11	(8.2)	**0.020**
LVEF 40–30%, (*n*, %)	61	(24.4)	35	(30.2)	26	(19.4)	**0.048**
LVEF <30%, (*n*, %)	129	(51.6)	44	(37.9)	85	(63.4)	**0.001**
LVEF not documented, (*n*, %)	23	-	5	-	18	-	-
VCI, cm (median, (IQR))	1.8	(1.5–2.2)	1.8	(1.4–2.2)	1.9	(1.6–2.2)	0.237
TAPSE, mm (median, (IQR))	15.0	(11.2–18.3)	16.0	(11.4–20.0)	14.5	(11.0–17.0)	0.368
**Cardiopulmonary resuscitation**							
OHCA, *n* (%)	104	(38.1)	37	(30.6)	67	(44.1)	**0.022**
IHCA, *n* (%)	47	(17.2)	13	(10.7)	34	(22.4)	**0.011**
Shockable rhythm, *n* (%)	76	(27.8)	34	(28.1)	42	(27.6)	0.932
Non-shockable rhythm, *n* (%)	197	(72.2)	87	(71.9)	110	(72.4)	0.932
ROSC, min (median, IQR)	15	(10–27)	12	(5–20)	17	(11–30)	**0.001**
**Respiratory status**							
Mechanical ventilation, *n* (%)	156	(57.1)	60	(49.6)	96	(63.2)	**0.024**
Duration of mechanical ventilation, days, (mean, (IQR))	2	(1–5)	2	(0–7)	2	(1–5)	0.250
PaO_2_/FiO_2_ ratio, (median, (IQR))	217	(133–353)	238	(146–367)	214	(120–336)	0.289
PaO_2_, mmHg (median, (IQR))	103	(77–165)	103	(78–161)	106	(77–169)	0.987
**Multiple organ support during ICU**							
Dosis norepinephrine on admission, µg/kg/min (median, (IQR))	0.1	(0.0–0.3)	0.1	(0.0–0.1)	0.2	(0.1–0.6)	**0.001**
Mechanical circulatory assist device, *n* (%)	25	(9.2)	5	(4.1)	20	(13.2)	**0.010**
**Baseline laboratory values**, (median, (IQR))							
pH	7.29	(7.19–7.37)	7.32	(7.24–7.37)	7.26	(7.15–7.36)	**0.002**
Lactate (mmol/L)	3.3	(1.7–7.2)	2.6	(1.6–4.0)	4.7	(2.4–10.3)	**0.001**
Serum sodium (mmol/L)	138	(136–141)	138	(136–140)	138	(136–141)	0.314
Serum potassium (mmol/L)	4.3	(3.8–4.9)	4.2	(3.7–4.8)	4.4	(3.9–5.0)	0.250
Serum creatinine (mg/dL)	1.48	(1.13–2.17)	1.31	(1.06–1.86)	1.59	(1.22–2.31)	**0.006**
Hemoglobin (g/dL)	12.4	(10.3–14.0)	12.4	(10.1–14.2)	12.4	(10.8–13.9)	0.913
WBC (10^6^/mL)	14.71	(10.47–18.88)	13.10	(9.68–17.64)	15.61	(12.20–19.73)	**0.002**
Platelets (10^6^/mL)	224	(171–274)	223	(163–285)	226	(177–266)	0.968
Cholesterol (mg/dL)	130	(98–170)	127	(95–170)	135	(102–166)	0.488
Triglycerides (mg/dL)	102	(71–143)	94	(65–136)	108	(82–153)	**0.026**
LDL (mg/dL)	87	(54–119)	87	(49–119)	88	(65–115)	0.786
HDL (mg/dL)	38	(29–49)	40	(32–49)	36	(28–49)	0.195
HbA_1c_ (%)	5.8	(5.4–6.9)	5.8	(5.3–6.8)	6.0	(5.6–7.6)	0.233
INR	1.17	(1.08–1.39)	1.13	(1.05–1.33)	1.20	(1.10–1.46)	**0.002**
D-dimer (mg/L)	9.69	(2.46–32.00)	5.49	(1.94–15.08)	17.76	(3.83–32.00)	**0.003**
AST (U/L)	129	(43–324)	109	(38–214)	167	(57–490)	**0.022**
ALT (U/L)	77	(32–186)	53	(30–130)	96	(35–295)	**0.013**
Bilirubin (mg/dL)	0.63	(0.43–0.99)	0.61	(0.41–0.95)	0.63	(0.46–1.00)	0.440
Albumin (g/L)	30.0	(25.5–33.9)	30.7	(27.6–34.4)	28.7	(23.8–33.0)	**0.005**
Troponin I (µg/L)	0.763	(0.164–6.154)	0.332	(0.087–2.494)	1.850	(0.344–12.431)	**0.001**
NT-pro BNP (pg/mL)	4387	(729–13,595)	3689	(454–12,343)	4462	(1110–13,946)	0.225
Procalcitonin (ng/mL)	0.30	(0.11–0.94)	0.31	(0.07–0.67)	0.28	(0.17–1.38)	0.529
CRP (mg/L)	13	(4–45)	12	(4–51)	15	(4–42)	0.665
**Primary endpoint**							
All-cause mortality at 30 days, *n* (%)	152	(55.7)	0	(0.0)	152	(100.0)	-
**Follow up data**, *n* (%)							
ICU time, days (median, (IQR))	4	(2–8)	4	(3–10)	3	(1–6)	**0.001**
Death ICU, *n* (%)	151	(55.3)	4	(3.3)	147	(96.7)	**0.001**

ADHF, acute decompensated heart failure; ALT, alanine aminotransferase; AST, aspartate aminotransferase; CRP, C-reactive Protein; CVR, cardiovascular risk; HDL, high-density lipoprotein; ICU, intensive care unit; HbA_1c_, glycated haemoglobin A1c; IHCA, in-hospital cardiac arrest; INR, international normalized ratio; IQR, interquartile range; LDL, low-density lipoprotein; NT-pro BNP, aminoterminal pro-B-type natriuretic peptide; OHCA, out-of-hospital cardiac arrest; ROSC, return of spontaneous circulation; TAPSE, tricuspid annular plane systolic excursion; VCI, Vena cava inferior; WBC, white blood cells. Level of significance *p* < 0.05. Bold type indicates statistical significance.

**Table 3:** Uni- and multivariable Cox regression analyses with regard to 30-day all-cause mortality.

Variables	Univariable			Multivariable		
	HR	95% CI	*p* Value	HR	95% CI	*p* Value
Age (years)	1.009	0.997–1.022	0.135	1.008	0.992–1.025	0.326
Sex	1.046	0.756–1.447	0.785	1.241	0.823–1.871	0.304
BMI (kg/m^2^)	1.017	0.987–1.048	0.265	0.999	0.962–1.037	0.951
Coronary artery disease	1.033	0.744–1.434	0.848	0.811	0.500–1.317	0.397
Congestive heart failure	0.937	0.671–1.309	0.703	0.998	0.574–1.738	0.995
Atrial fibrillation	0.976	0.693–1.374	0.890	0.802	0.494–1.302	0.372
Chronic kidney disease	1.015	0.726–1.420	0.929	0.895	0.504–1.589	0.705
Stroke	0.655	0.390–1.100	0.110	0.716	0.367–1.396	0.327
COPD	1.124	0.764–1.653	0.553	1.422	0.890–2.272	0.141
Acute myocardial infarction	1.653	1.199–2.281	**0.002**	2.089	1.205–3.620	**0.009**
ADHF	1.036	0.722–1.486	0.849	1.711	0.903–3.243	0.100
Acute kidney injury	1.798	1.216–2.658	**0.003**	1.721	0.985–3.004	0.056
Acute liver failure	0.995	0.608–1.627	0.983	0.666	0.342–1.298	0.232
Lactate (mmol/L)	1.127	1.093–1.161	**0.001**	1.134	1.087–1.183	**0.001**
Creatinine (mg/dL)	1.113	1.015–1.221	**0.023**	1.160	0.991–1.357	0.065
cTNI (µg/L)	1.002	1.001–1.003	**0.001**	1.001	1.000–1.003	**0.040**
High CVR	0.942	0.663–1.338	0.738	1.039	0.648–1.667	0.873

ADHF, acute decompensated heart failure; BMI, body mass index; COPD, chronic obstructive pulmonary disease; cTNI, cardiac troponin I; CVR, cardiovascular risk. Level of significance *p* < 0.05. Bold type indicates statistical significance.

**Table 4:** Uni- and multivariable Cox regression analyses with regard to 30-day all-cause mortality.

Variables	Univariable			Multivariable		
	HR	95% CI	*p* Value	HR	95% CI	*p* Value
Age (years)	1.009	0.997–1.022	0.135	1.016	1.000–1.032	**0.048**
Sex	1.046	0.756–1.447	0.785	1.157	0.797–1.680	0.444
BMI (kg/m^2^)	1.017	0.987–1.048	0.265	1.013	0.979–1.048	0.453
Lactate (mmol/L)	1.127	1.093–1.161	**0.001**	1.119	1.079–1.162	**0.001**
Creatinine (mg/dL)	1.113	1.015–1.221	**0.023**	1.101	0.985–1.231	0.090
Acute myocardial infarction	1.653	1.199–2.281	**0.002**	1.863	1.286–2.697	**0.001**
Arterial hypertension	0.906	0.638–1.287	0.582	0.679	0.433–1.065	0.092
Diabetes mellitus	1.213	0.881–1.671	0.237	1.112	0.751–1.646	0.596
Hyperlipidemia	0.718	0.516–0.998	**0.049**	0.801	0.536–1.195	0.277
Smoking	0.945	0.679–1.315	0.737	1.292	0.872–1.913	0.202

BMI, body mass index. Level of significance *p* < 0.05. Bold type indicates statistical significance.

## Data Availability

The datasets used and/or analyzed during the current study are available from the corresponding author upon reasonable request.

## Supplementary Materials

The following supporting information can be downloaded at: https://www.mdpi.com/article/10.3390/jcm12144870/s1. Table S1: Baseline characteristics; Table S2: Shock-related data, follow-up data and endpoints.
