# Supplementary material for: Effect of Cardiovascular Risk Factors on 30-Day All-Cause Mortality in Cardiogenic Shock
Source: J Clin Med. 2023 Jul 24;12(14):4870. doi: 10.3390/jcm12144870 (PMC10381971; doi:10.3390/jcm12144870)
Supplement: Supplementary file 1 [file jcm-12-04870-s001.zip › Table S1_supplementary.pdf]

Supplementary Table S1. Baseline characteristics.

	All patients (n=273)		Low CVR (n=77)		High CVR (n=196)		p value
**Age**, median; (IQR)	73	(63-81)	67	(53-78)	74	(64-81)	0.152
**Male sex,** n (%)	164	(60.1)	46	(59.7)	118	(60.2)	0.944
**Body mass index,** kg/m^2 (median, (IQR))	26.3	(24.2-30.0)	26.1	(23.4-28.4)	26.6	(24.2-30.4)	0.111
**Entry criteria,** (median, (IQR))							
Body temperature (°C)	36.0	(35.0-36.6)	35.8	(34.8-36.5)	36.0	(35.0-36.6)	**0.033**
Heart rate (bpm)	88	(71-109)	92	(71-106)	88	(71-110)	0.731
Systolic blood pressure (mmHg)	109	(92-130)	112	(98-135)	108	(89-127)	0.228
Respiratory rate (breaths/min)	20	(17-24)	19	(16-22)	20	(17-24)	0.066
**Cardiovascular risk factors,** n (%)							
Arterial hypertension	197	(72.2)	24	(31.2)	173	(88.3)	**0.001**
Diabetes mellitus Type 1	2	(0.7)	0	(0.0)	2	(1.0)	0.374
Diabetes mellitus Type 2	108	(39.6)	2	(2.6)	106	(54.1)	**0.001**
Hyperlipidaemia	187	(68.5)	18	(23.4)	169	(86.2)	**0.001**
Smoking	99	(36.3)	7	(9.1)	92	(46.9)	**0.001**
**Prior medical history,** n (%)							
Coronary artery disease	101	(37.0)	11	(14.3)	90	(45.9)	**0.001**
Congestive heart failure	96	(35.2)	11	(14.3)	85	(43.4)	**0.001**
Atrial fibrillation	87	(31.9)	11	(14.3)	76	(38.8)	**0.001**
Chronic kidney disease	93	(34.1)	9	(11.7)	84	(42.9)	**0.001**
Stroke	37	(13.6)	5	(6.5)	32	(16.3)	**0.033**
COPD	52	(19.0)	5	(6.5)	47	(24.0)	**0.001**
Liver cirrhosis	9	(3.3)	3	(3.9)	6	(3.1)	0.728
**Medication on admission**, n (%)							
ACE-inhibitor	92	(33.7)	11	(14.3)	81	(41.3)	**0.001**
ARB	48	(17.6)	8	(10.4)	40	(20.4)	**0.050**
Beta-blocker	135	(49.5)	19	(24.7)	116	(59.2)	**0.001**
ARNI	8	(2.9)	0	(0.0)	8	(4.1)	0.072
Aldosterone antagonist	41	(15.0)	5	(6.5)	36	(18.4)	**0.013**
Diuretics	117	(42.9)	14	(18.2)	103	(52.6)	**0.001**
ASA	78	(28.6)	14	(18.2)	64	(32.7)	**0.017**
P2Y12-inhibitor	23	(8.4)	0	(0.0)	23	(11.7)	**0.002**
Statin	119	(43.6)	8	(10.4)	111	(56.6)	**0.001**
Metformin	31	(11.4)	1	(1.3)	30	(15.3)	**0.001**
Sulfonylureas	3	(1.1)	1	(1.3)	2	(1.0)	0.843
GLP-1-RA	4	(1.5)	0	(0.0)	4	(2.0)	0.207
DPP-4-inhibitors	36	(13.2)	0	(0.0)	36	(18.4)	**0.001**
SGLT2-inhibitors	10	(3.7)	0	(0.0)	10	(5.1)	**0.043**
Insulin	47	(17.2)	1	(1.3)	46	(23.5)	**0.001**

ACE, angiotensin-converting-enzyme; ARB, angiotensin receptor blocker; ARNI, angiotensin receptor neprilysin inhibitor; ASA, acetylsalicylic acid; COPD, chronic obstructive pulmonary disease; CVR, cardiovascular risk; DPP-4-inhibitors, dipeptidyl peptidase-4 inhibitors; GLP-1-RA, glucagon-like peptide-1 receptor agonists; IQR, interquartile range; SGLT2-inhibitors, sodium-glucose cotransporter-2 inhibitors.
Level of significance $p<0.05$.
