# Supplementary material for: Effect of Cardiovascular Risk Factors on 30-Day All-Cause Mortality in Cardiogenic Shock
Source: J Clin Med. 2023 Jul 24;12(14):4870. doi: 10.3390/jcm12144870 (PMC10381971; doi:10.3390/jcm12144870)
Supplement: Supplementary file 1 [file jcm-12-04870-s001.zip › Table S2_supplementary.pdf]

Supplementary Table S2. Shock-related data, follow-up data and endpoints.

	**All patients** (n=273)		**Low CVR** (n=77)		**High CVR** (n=196)		**p value**
**Cause of CS,** n (%)							
Acute myocardial infarction	134	(49.1)	38	(49.4)	96	(49.0)	0.956
Arrhythmic	32	(11.7)	13	(16.9)	19	(9.7)	0.097
ADHF	67	(24.5)	11	(14.3)	56	(28.6)	**0.014**
Pulmonary embolism	15	(5.5)	8	(10.4)	7	(3.6)	**0.026**
Vitium	12	(4.4)	4	(5.2)	8	(4.1)	0.686
Cardiomyopathy	7	(2.6)	3	(3.9)	4	(2.0)	0.383
Pericardial tamponade	5	(1.8)	0	(0.0)	5	(2.6)	0.157
Aortic dissection	1	(0.4)	0	(0.0)	1	(0.5)	0.530
**Classification of CS,** n (%)							
Stage A	0	(0.0)	0	(0.0)	0	(0.0)	-
Stage B	6	(2.2)	3	(3.9)	3	(1.5)	0.230
Stage C	96	(35.2)	15	(19.5)	81	(41.3)	**0.001**
Stage D	20	(7.3)	4	(5.2)	16	(8.2)	0.397
Stage E	151	(55.3)	55	(71.4)	96	(49.0)	**0.001**
**Transthoracic echocardiography**							
LVEF >55%, (n, %)	28	(11.2)	8	(11.8)	20	(11.0)	0.863
LVEF 54-41%, (n, %)	32	(12.8)	8	(11.8)	24	(13.2)	0.765
LVEF 40-30%, (n, %)	61	(24.4)	21	(30.9)	40	(22.0)	0.145
LVEF <30%, (n, %)	129	(51.6)	31	(45.6)	98	(53.8)	0.245
LVEF not documented, (n, %)	23	-	9		14		-
VCI, cm (median, (IQR))	1.8	(1.5-2.2)	1.7	(1.4-2.0)	1.9	(1.6-2.2)	0.237
TAPSE, mm (median, (IQR))	15.0	(11.2-18.3)	15.0	(11.5-20.0)	15.0	(11.1-18.0)	0.697
**Cardiopulmonary resuscitation**							
OHCA, n (%)	104	(38.1)	43	(55.8)	61	(31.1)	**0.001**
IHCA, n (%)	47	(17.2)	12	(15.6)	35	(17.9)	0.654
Shockable rhythm, n (%)	76	(27.8)	35	(45.5)	41	(20.9)	**0.001**
Non-shockable rhythm, n (%)	197	(72.2)	42	(54.5)	155	(79.1)	**0.001**
ROSC, min (median, IQR)	15	(10-27)	15	(10-30)	15	(9-24)	**0.001**
**Respiratory status**							
Mechanical ventilation, n (%)	156	(57.1)	56	(72.7)	100	(51.0)	**0.001**
Duration of mechanical ventilation, days, (mean, (IQR))	2	(1-5)	2	(1-7)	2	(0-4)	0.250
PaO_2/FiO_2 ratio, (median, (IQR))	217	(133-353)	263	(110-391)	206	(133-335)	0.289
PaO_2, mmHg (median, (IQR))	103	(77-165)	111	(82-191)	101	(76-159)	0.987
**Multiple organ support during ICU**							
Dosis norepinephrine on admission, μg/kg/min (median, (IQR))	0.1	(0.0-0.3)	0.1	(0.0-0.5)	0.1	(0.0-0.2)	**0.001**
Mechanical circulatory assist device, n (%)	25	(9.2)	7	(9.1)	18	(9.2)	0.981
**Baseline laboratory values**, (median, (IQR))							
pH	7.29	(7.19-7.37)	7.28	(7.17-7.36)	7.29	(7.21-7.37)	**0.002**
Lactate (mmol/l)	3.3	(1.7-7.2)	3.4	(2.0-9.4)	3.3	(1.6-6.8)	**0.001**
Serum sodium (mmol/l)	138	(136-141)	138	(135-140)	138	(136-141)	0.314
Serum potassium (mmol/l)	4.3	(3.8-4.9)	4.0	(3.5-4.6)	4.4	(3.9-5.1)	0.250
Serum creatinine (mg/dl)	1.48	(1.13-2.17)	1.29	(1.06-1.68)	1.53	(1.21-2.46)	**0.006**
Hemoglobin (g/dl)	12.4	(10.3-14.0)	13.0	(11.6-14.5)	12.0	(10.2-13.8)	0.913
WBC (10^6/ml)	14.71	(10.47-18.88)	15.4	(10.1-19.3)	14.7	(10.7-18.6)	**0.002**
Platelets (10^6/ml)	224	(171-274)	224	(163-261)	224	(175-277)	0.968
Cholesterol (mg/dl)	130	(98-170)	140	(97-176)	125	(98-165)	0.488
Triglycerides (mg/dl)	102	(71-143)	95	(70-125)	108	(72-154)	**0.026**
LDL (mg/dl)	87	(54-119)	88	(57-109)	87	(54-120)	0.786
HDL (mg/dl)	38	(29-49)	47	(35-53)	36	(29-44)	**0.037**
HbA_{1c} (%)	5.8	(5.4-6.9)	5.4	(5.0-5.8)	6.0	(5.6-7.8)	0.233
INR	1.17	(1.08-1.39)	1.16	(1.08-1.32)	1.18	(1.08-1.40)	**0.002**
D-dimer (mg/l)	9.69	(2.46-32.00)	15.02	(5.78-32.00)	7.52	(1.92-24.96)	**0.003**
AST (U/l)	129	(43-324)	167	(101-467)	109	(38-265)	**0.022**
ALT (U/l)	77	(32-186)	99	(53.194)	69	(28-179)	**0.013**
Bilirubin (mg/dl)	0.63	(0.43-0.99)	0.63	(0.45-0.95)	0.63	(0.40-1.00)	0.440
Albumin (g/l)	30.0	(25.5-33.9)	29.0	(23.3-34.0)	30.0	(25.8-33.7)	**0.005**
Troponin I (μg/l)	0.763	(0.164-6.154)	1.118	(0.338-7.596)	0.706	(0.139-6.152)	**0.001**
NT-pro BNP (pg/ml)	4387	(729-13595)	2826	(213-14982)	4866	(1061-13595)	0.225
Procalcitonin (ng/ml)	0.30	(0.11-0.94)	0.28	(0.11-5.72)	0.31	(0.11-0.92)	0.529
CRP (mg/l)	13	(4-45)	7	(4-51)	14	(4-43)	0.665
**Primary endpoint**							
All-cause mortality at 30 days, n (%)	152	(55.7)	44	(57.1)	108	(55.1)	0.760
**Follow up data**, n (%)							
ICU time, days (median, (IQR))	4	(2-8)	4	(2-12)	4	(2-7)	**0.001**
Death ICU, n (%)	151	(55.3)	44	(57.1)	107	(54.6)	0.703

ADHF, acute decompensated heart failure; ALT, alanine aminotransferase; AST, aspartate aminotransferase; CRP, C-reactive Protein; CVR, cardiovascular risk; HDL, high-density lipoprotein; ICU, intensive care unit; HbA_{1c}, glycated haemoglobin A_{1c}; IHCA, in-hospital cardiac arrest; INR, international normalized ratio; IQR, interquartile range; LDL, low-density lipoprotein; NT-pro BNP, aminoterminal pro-B-type natriuretic peptide; OHCA, out-of-hospital cardiac arrest; ROSC, return of spontaneous circulation; TAPSE, tricuspid annular plane systolic excursion; VCI, Vena cava inferior; WBC, white blood cells.
Level of significance p<0.05. Bold type indicates statistical significance
